# Cerebral white matter hyperintensities on MRI and acceleration of epigenetic aging: the atherosclerosis risk in communities study

**DOI:** 10.1186/s13148-016-0302-6

**Published:** 2017-02-14

**Authors:** Abhay Raina, Xiaoping Zhao, Megan L. Grove, Jan Bressler, Rebecca F. Gottesman, Weihua Guan, James S. Pankow, Eric Boerwinkle, Thomas H. Mosley, Myriam Fornage

**Affiliations:** 1 Institute of Molecular Medicine, McGovern Medical School, University of Texas Health Science Center at Houston, 1825 Pressler Street, 77030 Houston, TX USA; 2 Division of Epidemiology, Human Genetics, and Environmental Sciences, School of Public Health, University of Texas Health Science Center at Houston, Houston, TX USA; 3 Department of Neurology, Johns Hopkins University School of Medicine, Baltimore, MD USA; 4 Division of Biostatistics, School of Public Health, University of Minnesota, Minneapolis, MN USA; 5 Division of Epidemiology & Community Health, School of Public Health, University of Minnesota, Minneapolis, MN USA; 6 Division of Geriatrics, School of Medicine, University of Mississippi Medical Center, Jackson, MS USA

**Keywords:** DNA methylation, Epigenetic clock, Epigenetic age acceleration, White matter hyperintensities, Cerebrovascular disease, Aging

## Abstract

**Background:**

Cerebral white matter hyperintensities (WMH) on magnetic resonance imaging (MRI) are part of the spectrum of brain vascular injury accompanying aging and are associated with a substantial risk of stroke and dementia. We investigated the association of cerebral WMH burden on MRI with a DNA methylation-based biomarker of aging, termed DNA methylation age acceleration, which represents the deviation of the DNA methylation-predicted age from the chronologic age.

**Results:**

In this cross-sectional observational study of 713 African-American participants of the Atherosclerosis Risk in Communities study, aged 51–73 years, estimates of predicted age were obtained based on two algorithms (Hannum et al. and Horvath) from DNA methylation measured using the Illumina HM450 array on genomic DNA extracted from blood. Age acceleration, calculated as the residual values from the regression of each of the predicted age measures onto the chronologic age, was significantly associated with WMH burden after accounting for chronologic age and sex, body mass index, systolic blood pressure, hypertension, diabetes, current smoking, and blood cell composition, and results were similar for either Hannum et al.- or Horvath-derived estimates (*P* = 0.016 and 0.026). An age acceleration increase by 1 year was associated with an increase of WMH burden by ~1 grade. To shed light on possible biological mechanisms underlying this association, we conducted a genome-wide association study of age acceleration and identified four loci harboring genes implicated in hemostasis, cell proliferation, protein degradation, and histone methylation. However, none of these loci were associated with WMH burden.

**Conclusions:**

In this population-based study of middle-aged to older African-American adults, we report an association between accelerated epigenetic aging and increased WMH burden, independent of known risk factors, including chronologic age. Additional studies are needed to clarify whether DNA methylation age reflects biological mechanisms implicated in the aging of the cerebral white matter.

**Electronic supplementary material:**

The online version of this article (doi:10.1186/s13148-016-0302-6) contains supplementary material, which is available to authorized users.

## Background

Cerebral small vessel disease is one of the most common degenerative vessel disorders of the aging brain. White matter hyperintensities (WMH) on brain magnetic resonance imaging (MRI) are typical markers of small vessel disease and are associated with an increased risk of stroke, cognitive and functional impairment, dementia, and death [1]. The prevalence of WMH increases with age, and advancing age is the strongest predictor of WMH severity [2–4]. Chronologic age, however, may not reliably gauge an individual’s rate of decline or changes related to the aging process. Thus, development of blood-based biomarkers of aging that could more accurately predict changes in brain morphology and function and their associated neurological outcomes is of long-standing interest.

DNA methylation is a key epigenetic mechanism of regulation of gene expression. Most human tissues and cell types exhibit profound changes in DNA methylation patterns with age [5]. Thus, recent studies have developed a novel biomarker of aging, termed “epigenetic clock”, based on DNA methylation levels at specific sites across the genome that are strongly correlated with chronologic age. Using whole blood DNA methylation array data from 656 subjects aged 19 to 101, Hannum et al. developed a predictor of age based on 71 CpG sites [6]. Similarly, using 82 publicly available DNA methylation array data sets comprising 51 healthy tissues and cell types across a range of chronologic ages, Horvath developed a multi-tissue predictor of age based on 353 CpG sites [7]. The deviation of the DNA methylation-predicted age from the chronologic age, defined as “age acceleration”, is used as an index of “biological” aging.

Recent studies have shown significant associations between blood DNA methylation-derived measures of accelerated aging and all-cause mortality [8], as well as physical and cognitive fitness late in life [9]. Here, we estimated blood DNA methylation age in African-Americans from the Atherosclerosis Risk in Communities (ARIC) study using, both, Horvath and Hannum et al. methodologies, and examined the cross-sectional association between cerebral white matter hyperintensities on MRI and measures of accelerated epigenetic aging.

## Methods

### Study design and participants

The ARIC study is a prospective population-based study of atherosclerosis and clinical atherosclerotic diseases. At its inception (1987–1989), 15,792 men and women, including 4266 African-American participants aged 45 to 64, were recruited from probability samples in four US communities: Suburban Minneapolis, MN; Washington County, MD; Forsyth County, NC; and Jackson, MS (African-Americans only) [10]. Four additional examinations (visit 2: 1990–1992; visit 3: 1993–1995; visit 4: 1996–1998; and visit 5: 2011–2013) have been completed. During the first 2 years of the third ARIC examination, participants aged 55 and older from the Forsyth County and Jackson sites were invited to undergo a cranial MRI. A total of 1920 participants, including 955 African-Americans had usable MRI data. All methods were approved by the institutional review board at each field center and coordinating center, and written informed consent was obtained from the participants.

### MRI protocol and phenotyping

Details of the MRI scanning and the image interpretation protocols used for this study have been published [11, 12]. General Electric (General Electric Medical Systems) or Picker (Picker Medical Systems) 1.5-T scanners were used for the MRI examination. The scanning protocol included a series of sagittal T1-weighted scans and axial proton density-weighted, T2-weighted, and T1-weighted scans with 5-mm thickness and no interslice gaps. Images were interpreted directly from a PDS-4 digital workstation consisting of four 1024 × 1024-pixel monitors capable of displaying all 96 images simultaneously. WMH were estimated as the relative total volume of periventricular and subcortical white matter signal abnormality on proton density-weighted axial images by visual comparison with eight templates that successively increased from barely detectable white matter abnormalities (grade 1) to extensive, confluent abnormalities (grade 8). Individuals with no white matter abnormalities received grade 0, and those with abnormalities worse than grade 8 received grade 9.

### DNA methylation analysis

DNA methylation analysis was conducted with the Infinium HumanMethylation450 BeadChip (HM450) array (Illumina Inc., San Diego, CA) on genomic DNA extracted from blood samples collected at ARIC visit 2 or 3. Assay were performed on 2879 African-American participants who had not restricted use of their DNA and for whom at least 1 μg of DNA and genome-wide genotyping data were available.

Details of assay and QC procedures have been previously published [13]. Briefly, genomic DNA was treated with sodium bisulfite using the EZ-96 DNA methylation kit (Zymo Research Corporation, Irvine, CA) following the manufacturer’s protocol. Bisulfite converted DNA was amplified, enzymatically fragmented, purified, and hybridized to the HM450 array in accordance with the manufacturer’s directions. Methylation typing at 485,577 CpG sites was performed using GenomeStudio 2011.1 (Illumina Inc., San Diego, CA). Methylation level for each probe was derived as a beta value representing the fractional level of methylation at that location. Quality control analysis was performed using the wateRmelon R package [14]. Probe data were excluded if they had a low detection rate (<95% at *P* < 0.01) and a high missing rate (greater than 1% across all samples). Sample data were excluded based on the following criteria: (1) greater than 5% missing values across all probes; (2) possible gender mismatch based on principal component analysis; and (3) genotype mismatch based on 24 SNPs present on the HM450 array.

A total of 713 participants with, both, a DNA methylation measure and a brain MRI scan are included in these analyses. For each participant, DNA methylation age was estimated from the background-subtracted beta values processed in GenomeStudio. Imputation of missing beta values and data normalization were performed using R codes implemented by Horvath. Specifically, non-normalized beta values were uploaded, and data normalization option was selected. Two estimates of DNA methylation age were derived: The Horvath predicted age based on 353 CpG probes was generated using the online calculator; and the Hannum et al. predicted age based on 71 probes was derived using the regression weights supplied by the authors [6]. Age acceleration estimates were calculated as the residual values from the regression of each of the predicted age measures onto the chronologic age at the time of blood collection.

### Statistical analysis

To reduce the skewness of its distribution, WMH burden was expressed as the natural log-transformed WM grade (log(WMH + 1)). Linear regression models were used to estimate the association of WMH burden (log(WMH + 1)) with either Horvath or Hannum et al. estimate of age acceleration adjusting for covariates. Covariates were measured at ARIC visit 3 except for blood cell composition, which were estimated from the DNA methylation data as described elsewhere [15, 16]. Model 1 adjusted for age at brain MRI and sex. Model 2 adjusted for age at brain MRI, sex, body mass index, systolic blood pressure, hypertension, diabetes, and current smoking. Model 3 adjusted for covariates in model 2 and blood cell composition (estimated proportions of CD8 and CD4 T cells, natural killer cells, plasma blasts, monocytes, and granulocytes were derived from Horvath algorithm).

To examine whether estimates of age acceleration were associated with greater WMH severity, WMH was classified into three categories: no WMH (grade 0); low WMH burden (grade = 1–4); and high WMH burden (grade > =5). Multinomial logistic regression was used to estimate the association of WMH severity categories with estimates of age acceleration adjusting for covariates. The covariate adjustment scheme was the same as that described for the linear models. Statistical analyses were performed using the SAS software v9.4.

### Genome-wide association analysis of age acceleration and association of age acceleration loci with WMH

Genome-wide genotyping was conducted at the Broad Institute using the Affymetrix 6.0 SNP Array on 3207 African-American participants. Of these, 336 were removed in data cleaning procedures, which included an insufficient call rate, sex mismatch, discordance with previously genotyped markers, first-degree relative of an included individual, and genetic outlier based on allele sharing and principal components analyses. Imputation was performed on the QCed data in two steps: (1) pre-phasing with ShapeIt (v1.r532) and (2) imputation with IMPUTE2 using the 1000 Genomes Phase I v3 reference panel. Measured SNPs used for imputation were restricted to have MAF > 0.01, >95% call rate, and Hardy Weinberg Equilibrium *P* > 1 × 10^−6^. After frequency and genotyping pruning, there were 806,416 SNPs in the final set used for the imputation.

Statistical analyses modeled the association between the number of minor alleles at each SNP and each measure of age acceleration trait using linear regression, adjusting for chronologic age, gender, study site, blood cell composition and 10 PCs. A *P* value less than 5 × 10^−8^ was considered statistically significant. There were 2216 ARIC participants with both DNA methylation and genome-wide genotyping included in the analyses. Significant epigenetic age acceleration loci were, then, evaluated for their association with WMH using the largest published GWAS of WMH to date [17].

### Epigenome-wide association analysis of WMH burden

We examined the association of individual CpG probes with WMH burden in the 713 individuals with DNA methylation and brain MRI data. The association between methylation scores at each CpG site and log(WMH + 1) was modeled using linear mixed models, adjusting for age at brain MRI, gender, study site, visit, BMI, smoking, blood cell composition, systolic blood pressure, diastolic blood pressure, and technical variables (plate ID, chip ID and chip row). For this analysis, beta values normalized using the BMIQ procedure [18] were used. Probes with poor reliability in our technical replicate analyses [19], probes containing known SNPs, and non-specific probes were excluded [20, 21]. A *P* value less than 1 × 10^−7^, which corresponds to a Bonferroni correction to the total number of CpGs tested of the *P* = 0.05 threshold, was considered statistically significant.

## Results

Selected characteristics of the participants are shown in Table 1. In this group of 713 African-Americans aged 51–73 years, 579 (81%) had a WMH grade >0. Pearson correlations between measures of chronologic age, estimates of DNA methylation age, and estimates of age acceleration are shown in Table 2. On average, the Horvath predicted age was lower than the chronologic age, while the Hannum et al. predicted age was higher (mean difference: −1.5 vs. 3.4 years). The Pearson correlation between the chronologic age of the subjects and the Horvath predicted age was *ρ* = 0.51, while the correlation with the Hannum et al. predicted age was *ρ* = 0.62. The correlation between the two sets of predicted age values was *ρ* = 0.69. Age acceleration estimates were not correlated with chronologic age measures. Because a subset of the subjects (*N* = 604) had their blood collection earlier than their MRI scan, we also calculated the Pearson correlation between the participants’ chronologic age at which blood was collected and the chronologic age at the time of the MRI scan, which was very high (*ρ* = 0.96). For this subset of subjects, blood collection was on average 2.94 ± 0.28 years earlier than brain MRI examination.

**Table 1:** Mean (SD) or proportion for selected characteristics of the study sample at the MRI examination (ARIC Visit 3)

Number	713
Women, *N* (%)	456 (64)
Chronologic age at MRI visit, years	61.8 (4.5)
Chronologic age at DNA methylation measure, years	59.3 (4.6)
Predicted methylation age (Horvath), years	57.7 (6.5)
Predicted methylation age (Hannum et al.), years	62.7 (7.1)
Age acceleration (Horvath), years	0.18 (5.59)
Age acceleration (Hannum et al.), years	−0.49 (5.56)
White matter grade	1.42 (1.33)
Systolic blood pressure, mmHg	134.4 (21.5)
Diastolic blood pressure, mmHg	75.5 (10.5)
Body mass index, kg/m^2^	29.6 (5.3)
Hypertension, %	382 (54.3)
Diabetes, %	159 (22.5)
Current smoking, *N* (%)	137 (19.3)

**Table 2:** Pearson correlation between measures of chronologic age, estimates of DNA methylation age, and estimates of age acceleration

	Age at DNA methylation measure	Age at MRI scan	Horvath DNA methylation age	Hannum et al. DNA methylation age	Horvath age acceleration	Hannum et al. age acceleration
Age at blood collection	1	0.96*	0.50*	0.62*	−0.008	−0.006
Age at MRI scan		1	0.51*	0.62*	0.02	0.02
Horvath DNA methylation age			1	0.69*	0.86*	0.48*
Hannum et al. DNA methylation age				1	0.43*	0.78*
Horvath age acceleration					1	0.56*
Hannum et al. age acceleration						1

*P < 0.0001

Chronologic age and DNA methylation age were significantly associated with WMH burden (Table 3). Chronologic age explained ~6–8% of the variance in WMH grade (on the log scale) adjusting for known risk factors. The proportion of variance explained was generally higher for Hannum et al. DNA methylation age than for Horvath DNA methylation age (~5 to 8% vs. 3.5 to 5%), but results from the two estimates were more comparable after adjustment for blood cell composition (Table 3, model 3).

**Table 3:** Association of WMH burden with chronologic age or estimates of DNA methylation age

	Model 1		Model 2		Model 3	
	*R* ^2^ (%)	*P* value	*R* ^2^ (%)	*P* value	*R* ^2^ (%)	*P* value
Chronologic age^a^	8.06	<0.0001	7.71	<0.0001	5.62	<0.0001
Horvath DNA methylation age	4.74	<0.0001	5.00	<0.0001	3.51	<0.0001
Hannum et al. DNA methylation age	7.84	<0.0001	6.86	<0.0001	4.72	<0.0001

Model 1: adjusted for age at brain MRI^a^ and sex. Model 2: adjusted for age at brain MRI^a^, sex, body mass index, systolic blood pressure, hypertension, diabetes, and current smoking. Model 3: adjusted for covariates in model 2 and white blood cell composition

^a^Models estimating the association of chronologic age with WMH are not adjusted for age at brain MRI

Age acceleration, defined as the residual value from the regression of the predicted DNA methylation age onto the chronologic age (at the time of DNA methylation assay), was significantly associated with WMH burden. An age acceleration increase by 1 year was associated with an increase of WMH burden by ~1 grade (0.007 on the log scale) (Table 4). The association of Hannum et al. age acceleration was slightly stronger than that of Horvath, although adjustment for blood cell composition yielded similar results.

**Table 4:** Association between WMH burden and age acceleration estimates

	Horvath predictor			Hannum et al. predictor		
	Beta (SD)^a^	*P* value	% variance explained	Beta (SD)^a^	*P* value	% variance explained
Model 1	0.007 (0.003)	0.0287	0.61	0.011 (0.003)	0.0005	1.51
Model 2	0.007 (0.003)	0.0134	0.74	0.009 (0.003)	0.0044	1.12
Model 3	0.007 (0.003)	0.0258	0.60	0.009 (0.003)	0.0162	0.69

Model 1: adjusted for age at brain MRI and sex. Model 2: adjusted for age at brain MRI, sex, body mass index, systolic blood pressure, hypertension, diabetes, and current smoking. Model 3: adjusted for covariates in model 2 and white blood cell composition

*SD* standard deviation

^a^WMH grade unit

When WMH burden was classified into categories (no WMH, low, and high burden), there was a significant association of WMH severity with age acceleration using either the Horvath or Hannum et al. predictor and accounting for age, sex, and known risk factors (Table 5). Further adjusting for blood cell composition did not appreciably change these results. Compared to those with no WMH burden (grade = 0), those with high WMH burden (grade = 5 or greater) had a significantly greater age acceleration by either predictor (*P* = 0.02, Horvath; *P* = 0.03, Hannum et al.). By contrast, there was no association of age acceleration with low WMH burden.

**Table 5:** Association between age acceleration and WMH categories

		Horvath predictor			Hannum et al. predictor		
		OR (95% CI)	*P* value	*P* trend	OR (95% CI)	*P* value	*P* trend
Model 1	No WMH	1 (Ref)		0.08	1 (Ref)		0.08
	Low WMH burden	1.01 (0.98–1.05)	0.40		1.03 (0.99–1.01)	0.10	
	High WMH burden	1.08 (1.01–1.17)	0.03		1.12 (1.04–1.21)	0.002	
Model 2	No WMH	1 (Ref)		0.03	1 (Ref)		0.02
	Low WMH burden	1.01 (0.98–1.05)	0.44		1.02 (0.99–1.06)	0.20	
	High WMH burden	1.11 (1.02–1.20)	0.01		1.15 (1.03–1.21)	0.007	
Model 3	No WMH	1 (Ref)		0.06	1 (Ref)		0.07
	Low WMH burden	1.02 (0.98–1.05)	0.33		1.03 (0.99–1.08)	0.13	
	High WMH burden	1.11 (1.02–1.20)	0.02		1.11 (1.01–1.21)	0.03	

No WMH (grade 0), *N* = 134; low WMH (grades 1–4), *N* = 546; high WMH (grades > =5), *N* = 33. Model 1: adjusted for age at brain MRI and sex. Model 2: adjusted for age at brain MRI, sex, body mass index, systolic blood pressure, hypertension, diabetes, and current smoking. Model 3: adjusted for covariates in model 2 and white blood cell composition

*OR* odds ratio, *95% CI* 95% confidence interval, *Ref* reference, *P trend P* value of test for trend

**Fig. 1:**
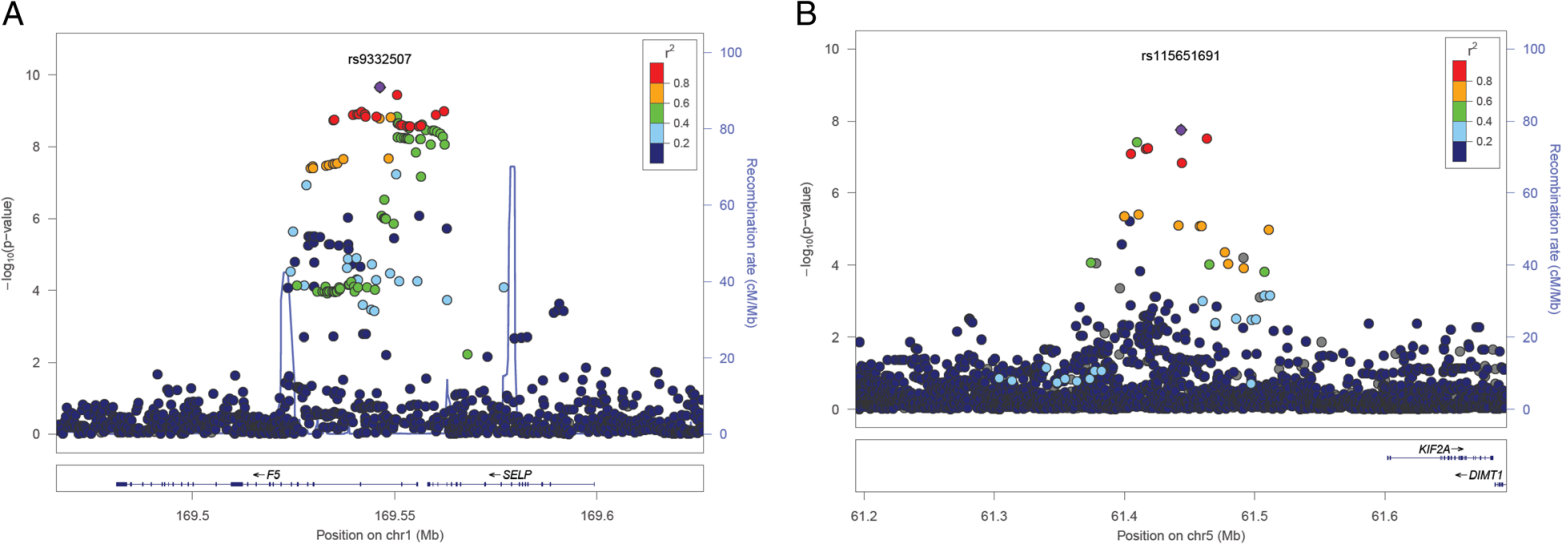
Regional plots of association between SNPs at the chr1q14 (**a**) and chr5q12 (**b**) loci with age acceleration derived from Hannum et al. estimates of DNAm age

**Fig. 2:**
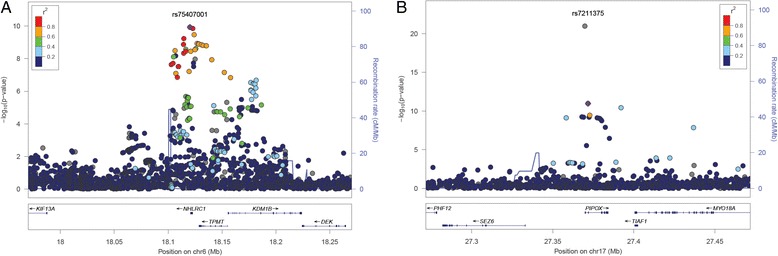
Regional plots of association between SNPs at the chr6p22 (**a**) and chr17q11 (**b**) loci with age acceleration derived from Horvath estimates of DNAm age

To further examine whether the relationship between WMH and epigenetic age acceleration reflects biological mechanisms implicated in the aging of the cerebral white matter, we sought to identify genetic loci influencing age acceleration using genome-wide association analysis in 2216 African-American ARIC participants with DNA methylation and genome-wide genotyping data. For age acceleration derived from the Hannum et al. algorithm, we identified two regions with genome-wide significant SNPs: 1q24 (top SNP: rs9332507, *P* = 2.2 × 10^−10^) and 5q12 (top SNP: rs115651691, *P* = 1.7 × 10^−8^). For age acceleration derived from the Horvath algorithm, we identified two regions with genome-wide significant SNPs: 6p22 (top SNP: rs75407001; *P* = 1.0 × 10^−11^) and 17q11 (top SNP: chr17:2736978 I/D, *P* = 3.5 × 10^−23^). The chr1q24 locus contained 62 SNPs with *P* < 5 × 10^−8^ (Fig. 1a). The top SNP rs9332507 (MAF = 0.087) lies within an intron of the *F5* gene, which encodes coagulation factor V. Of the 62 chr1q24 SNPs associated with Hannum et al. age acceleration, 12 SNPs, all in high to moderate linkage disequilibrium (LD), were also nominally associated with Horvath age acceleration (*P* < 0.05). The chr5q12 contained three SNPs with *P* < 5 × 10^−8^ (Fig. 1b). The three SNP, all in high to moderate LD, lie near *KIF2A*, encoding the kinesin heavy chain member 2A. All three SNPs were also nominally associated with Horvath age acceleration (*P* ≤ 0.001). The chr6q22 locus contained 32 SNPs with *P* < 5 × 10^−8^ (Fig. 2a). The top SNP rs75407001 (MAF = 0.076) lies 425 bp downstream of *NHLRC1*, which encodes an E3 ubiquitin ligase. None of the chr6q22 was also associated with Hannum et al. age acceleration. The chr17q11 locus contained 18 SNPs with *P* < 5 × 10^−8^ (Fig. 2b). The top SNP chr17:2736978 (MAF = 0.046) is an indel located 138 bp upstream of *PIPOX*, encoding pipecolic acid oxidase. Only one of the 18 SNPs in the chr17q11 region (rs140625670) was also nominally associated with Hannum et al. age acceleration (*P* = 0.01). This SNP was located in an intron of *NUFIP2*, encoding nuclear fragile X mental retardation protein interacting protein 2.

We then examined the association of selected SNPs in each locus with WMH burden in middle-aged to elderly individuals from population-based cohorts of the NeuroCHARGE consortium, including 17,936 participants of European ancestry and 1943 African-Americans [17]. From each region, we selected the SNP that was most significantly associated with both measures of age acceleration (chr1q24, 5q12, and chr17q11) or the topSNP (chr6q22). There was no evidence of an association of age acceleration loci with WMH burden (Table 6). Although rs6703462 on chr1q24 was nominally associated with WMH in Europeans, the direction of association was not consistent with the observed relationship of an increased age acceleration with an increased WMH burden.

**Table 6:** Association between selected SNPs in each age acceleration locus and WMH burden in 17,936 middle-aged to elderly participants from the NeuroCHARGE consortium

SNP	Chromosome	Coded allele	Coded allele frequency (AA/EA)	*P* value (direction) of association with Hannum age acceleration	*P* value (direction) of association with Horvath age acceleration	*P* value (direction) of association with WMH burden	
						European	African-American
rs6703462	1q24	T	0.16/0.08	1.5 × 10^−9^ (+)	0.0097 (+)	0.029 (−)	0.20 (+)
rs4635894	5q12	T	0.06/0.07	3.0 × 10^−8^ (+)	0.0011 (+)	0.55 (+)	0.52 (−)
rs75407001	6p22	T	0.08/0.04	0.26 (−)	1 × 10^−11^ (−)	0.19 (−)	0.68 (+)
rs140625670	17q11	C	0.01/NA	0.01 (−)	4.3 × 10^−8^ (−)	NA	0.29 (−)

**Fig. 3:**
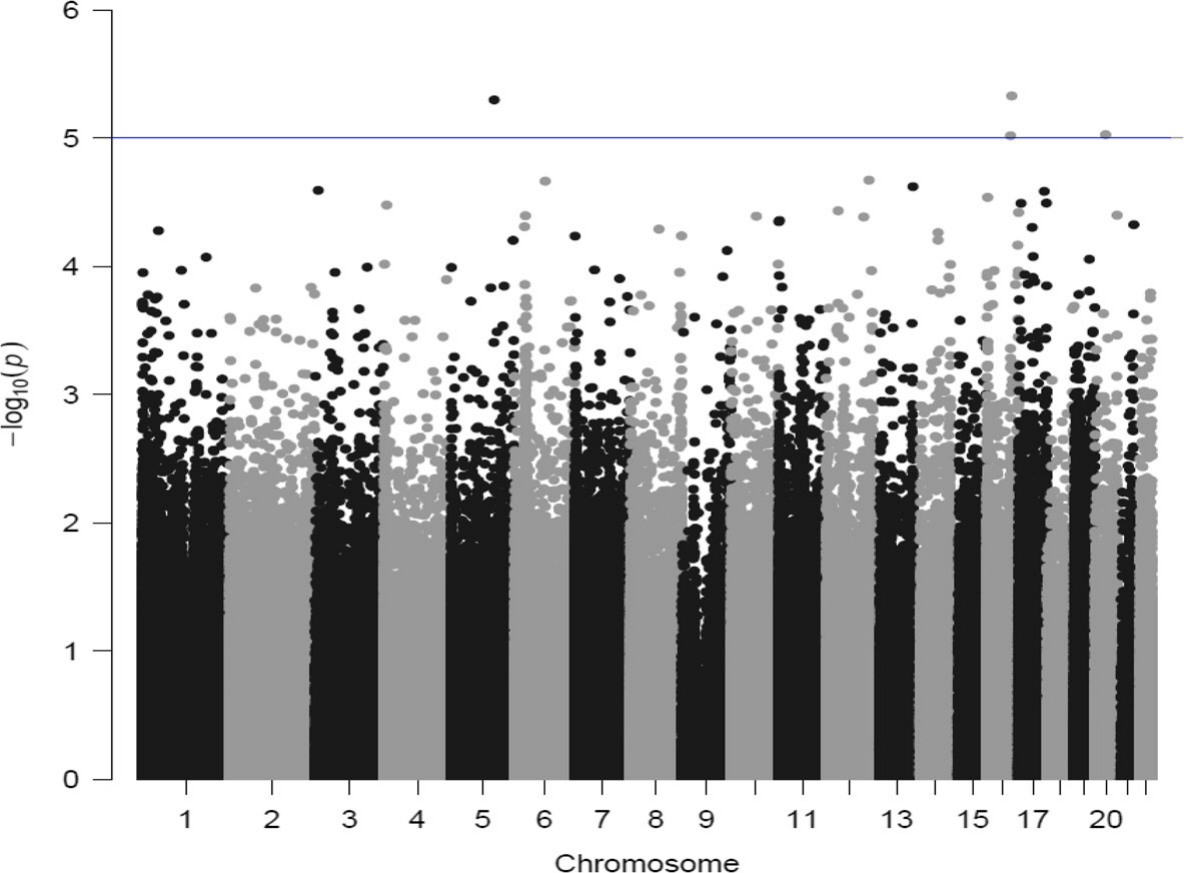
Manhattan plot of epigenome-wide association (EWAS) of WMH burden in ARIC African-Americans. *P* values of association of DNA methylation sites with WMH burden are plotted against chromosomal location. The threshold for suggestive evidence of association (*P* < 10^−5^) is shown

Finally, we examined the association of WMH burden with methylation levels of individual CpG probes, first, among the 71 Hannum et al. and 353 Horvath CpGs probes used to estimate DNA methylation (DNAm) age and then genome-wide. Figure 3 shows the Manhattan plot of the epigenome-wide association study (EWAS) of WMH burden in ARIC African-Americans. No probe reached the Bonferroni corrected threshold of significance either in targeted analyses or genome-wide. A more complete description of these results is available as Additional file 1.

## Discussion

In this population-based study of middle-aged to older African-American adults, we report evidence of an association between accelerated epigenetic aging assessed in blood and increased WMH burden and severity, independent of known risk factors, including chronologic age. On average, an increase in WMH burden by one grade was associated with an older epigenetic age by ~1 year. These results are consistent with those of a prior study [22] reporting an association of WMH with leukocyte telomere length, another biomarker of aging, independent of known risk factors; and raise the possibility that mechanisms of accelerated biological aging may be implicated in WMH pathophysiology.

We applied two distinct predictors of DNAm age. While the association of DNAm age acceleration with WMH appeared generally stronger using the blood-based predictor of Hannum et al., adjustment for blood cell composition yielded comparable estimates than those of the multi-tissue predictor of Horvath, which is more robust to the tissue type from which DNA methylation age is derived. Consistency of findings between the two distinct predictors suggest that the relationship between epigenetic age acceleration and cerebral WMH burden does not depend on specific epigenetic signatures but likely reflects the association of WMH with a general biomarker of “biological” aging.

Whether the relationship between DNAm age and WMH reflects biological mechanisms involved in the aging of the white matter remains to be determined. A previous study by Marioni et al. suggested that about 40% of interindividual variation in age acceleration is due to genetic factors and this heritability estimate was similar for both Hannum et al.- and Horvath-derived measures [8]. These data suggest that DNAm age acceleration may capture, in part, biological differences that affect the rate of age-related decline. We, thus, conducted a genome-wide association analysis of age acceleration and identified four loci, which were largely distinct for the two measures. The two loci identified for Hannum et al.-derived age acceleration encompassed genes involved in hemostasis (*F5*, *SELP*) and cell cycle and proliferation (*KIF2A*) and were only nominally associated with the Horvath-derived measure of age acceleration. Two distinct loci were identified for Horvath-derived age acceleration, which encompassed genes involved in protein degradation (*PIPOX*, *NHLRC1*), detoxification (*TPMT*) and histone methylation (*KDM1B*). None of the SNPs associated with age acceleration were significantly associated with WMH in the largest GWAS of WMH published to date [17], suggesting that loci implicated in age acceleration do not significantly contribute to WMH pathophysiology.

No individual CpG probe reached genome-wide significance for association with WMH in this cohort of middle-aged to older African-American adults. Suggestive associations include cg10262662 (*P* = 4.7 × 10^−6^) mapping to the 3′ UTR of the *VAC14* gene, which encodes a component of a trimolecular complex that tightly regulates the level of phosphatidylinositol 3,5-bisphosphate (PI(3,5)P2). Mice lacking the *Vac14* gene show a significant decrease in cellular levels of phosphatidylinositol polyphosphates, including PI(3,5)P2, and exhibit significant neurodegeneration [23]. Mice carrying a mutation in the *Vac14* gene that results in significantly reduced PI(3,5)P2, show an accumulation of membrane-bounded vesicles in neurons of the central nervous system, an early feature of neurodegeneration, astrocytosis, and defective autophagy [24]. Future studies will include additional samples to identify robust associations with WMH.

Several limitations of our study must be acknowledged. First, DNA methylation was measured in blood. It is not clear whether DNAm age estimated from blood can serve as a proxy for that of brain, a likely more relevant tissue for cerebrovascular disease, or whether DNAm age estimates derived from more specific and relevant tissues would provide more accurate estimates of age acceleration and, thus, more robust associations with disease risk. Second, our analyses were cross-sectional, and DNAm was measured as a single time point. Thus, we are unable to examine whether change in DNAm age is associated with change in WMH burden, which would strengthen our findings. Third, we were unable to establish whether the association between epigenetic aging and WMH burden reflects biological mechanisms implicated in aging-related injury to the white matter. Indeed, there was no association between genetic loci influencing age acceleration and WMH burden in the largest GWAS published to date [17]. However, it is worth pointing out that our GWAS of age acceleration was performed in a single study of African-Americans. While the published GWAS of WMH includes a large sample of Europeans, the African-American sample was comparatively small and, thus, may be insufficiently powered to uncover race-specific relationships. Additional studies will be needed to clarify these relationships. Finally, we did not independently replicate the described association between DNAm age acceleration and WMH. Race/ethnicity has been recently associated with epigenetic age acceleration in blood, with African-Americans exhibiting significantly lower age acceleration than whites, especially at older ages [25]. Additional studies, preferably in African-Americans, will be needed to corroborate our findings.

## Conclusions

Overall, our study shows that epigenetic aging in blood is associated with WMH burden, suggesting that the epigenetic clock may represent a novel molecular biomarker of vascular and neurodegenerative aging of the brain. Future studies in expanded samples will examine whether the relationship between epigenetic age acceleration and WMH burden reflects biological mechanisms implicated in the aging of the cerebral white matter.

## Additional file

Additional file 1: Top 100 CpGs from the epigenome-wide association study (EWAS) of WMH burden in ARIC African-Americans. Association *P*-value, effect size, and standard error for each probe are shown as well as location(chromosome, position) and probe type. (PDF 195 kb)

## Abbreviations

ARIC: Atherosclerosis Risk in Communities
DNAm: DNA methylation
EWAS: Epigenome-wide association study
GWAS: Genome-wide association study
MRI: Magnetic resonance imaging
WMH: White matter hyperintensities
