# Supplementary material for: Cerebral white matter hyperintensities on MRI and acceleration of epigenetic aging: the atherosclerosis risk in communities study
Source: Clin Epigenetics. 2017 Feb 14;9:21. doi: 10.1186/s13148-016-0302-6 (PMC5310061; doi:10.1186/s13148-016-0302-6)
Supplement: Additional file 1: — Top 100 CpGs from the epigenome-wide association study (EWAS) of WMH burden in ARIC African-Americans. Association P-value, effect size, and standard error for each probe are shown as well as location(chromosome, position) and probe type. (PDF 195 kb) [file 13148_2016_302_MOESM1_ESM.pdf]

CPG_Labels	P_value	effect_size	std_error	Infinium_Design_Type	CHR	MAPINFO	Strand
cg10262662	4.70E-06	-0.0234	0.0051	II	16	70721696	F
cg11616701	5.05E-06	-0.0153	0.0033	II	5	122950706	F
cg11845410	9.41E-06	0.0205	0.0046	II	20	30413603	F
cg04607024	9.60E-06	-0.0378	0.0085	II	16	67696204	F
cg14917044	2.12E-05	-0.0160	0.0037	I	12	122257870	F
cg24605950	2.15E-05	0.0789	0.0184	II	6	87222174	F
cg26531551	2.38E-05	-0.0103	0.0024	I	13	113702436	F
cg00498591	2.55E-05	0.0083	0.0020	I	3	9904557	F
cg06708315	2.59E-05	-0.0100	0.0024	II	17	73689641	F
cg06664900	2.88E-05	-0.0241	0.0057	II	16	2073792	R
cg14852540	3.20E-05	-0.0129	0.0031	II	17	79683076	R
cg14372120	3.21E-05	-0.0153	0.0037	II	17	7258501	R
cg05779630	3.31E-05	0.0325	0.0078	II	4	7473106	R
cg10931853	3.67E-05	-0.0489	0.0118	I	12	34548115	F
cg01973164	3.78E-05	-0.0119	0.0029	II	16	89972593	R
cg26194514	3.98E-05	-0.0259	0.0063	II	20	62575851	F
cg11402847	4.02E-05	-0.0056	0.0014	II	6	31475203	F
cg19616532	4.04E-05	-0.0110	0.0027	II	10	71869135	F
cg27081585	4.11E-05	-0.0124	0.0030	II	12	107280558	R
cg07804260	4.38E-05	-0.0220	0.0053	II	11	2397977	F
cg01669186	4.47E-05	0.0078	0.0019	I	11	430919	R
cg26721908	4.72E-05	-0.0209	0.0051	II	21	47610096	R
cg17164315	4.89E-05	-0.0564	0.0138	I	6	28607804	F
cg02385230	4.95E-05	-0.0119	0.0029	II	17	38605309	F
cg15708373	5.10E-05	-0.0025	0.0006	II	8	81397789	R
cg15198148	5.24E-05	-0.0946	0.0232	II	1	46858748	R
cg00807877	5.45E-05	-0.0495	0.0122	II	14	70095319	R
cg02390569	5.78E-05	-0.0225	0.0055	II	8	145624983	R
cg06469440	5.80E-05	-0.0171	0.0042	II	7	1000336	F
cg00785892	6.23E-05	-0.0124	0.0031	II	14	70701435	R
cg14126493	6.26E-05	0.0144	0.0036	I	5	176831297	R
cg06969933	6.82E-05	-0.0093	0.0023	II	16	88099740	F
cg14521987	7.52E-05	0.0092	0.0023	II	9	129236500	F
cg02173360	8.34E-05	-0.0443	0.0112	I	17	41312467	F
cg01079738	8.48E-05	0.0408	0.0103	I	1	182922427	R
cg13834567	8.80E-05	0.0888	0.0225	II	19	42817981	R
cg22245862	9.61E-05	-0.0204	0.0052	II	4	942544	R
cg04913109	9.61E-05	-0.0156	0.0040	I	10	134538898	R
cg18970434	9.66E-05	-0.0188	0.0048	II	14	106067003	F
cg25349034	1.02E-04	0.0133	0.0034	II	3	149265300	F
cg18196463	1.02E-04	-0.0050	0.0013	I	5	1069235	R
cg03894119	1.06E-04	-0.0060	0.0015	I	7	57247873	R
cg27414885	1.07E-04	0.0059	0.0015	II	1	112298991	F
cg26980987	1.08E-04	-0.0151	0.0039	II	16	20754216	F
cg05253716	1.08E-04	-0.0112	0.0029	II	12	129299332	R
cg03568793	1.09E-04	-0.0088	0.0023	II	16	89620236	F

cg08993582	1.11E-04	0.0061	0.0016	I	3	57678673	F
cg11240431	1.11E-04	-0.0153	0.0039	II	8	140971271	R
cg25037677	1.12E-04	-0.0202	0.0052	II	1	2437047	R
cg09414620	1.13E-04	0.0350	0.0090	II	16	1213469	F
cg11300341	1.16E-04	-0.0119	0.0031	II	17	17689562	R
cg00364644	1.18E-04	-0.0141	0.0036	I	16	88234627	R
cg25226711	1.18E-04	-0.0493	0.0127	II	11	1297385	F
cg03948744	1.20E-04	0.0180	0.0046	I	16	122563	R
cg13632058	1.20E-04	0.0210	0.0054	II	9	117267392	R
cg05465955	1.21E-04	-0.0322	0.0083	I	17	40715236	F
cg27039821	1.22E-04	-0.0070	0.0018	I	14	101340965	F
cg15992868	1.24E-04	-0.0082	0.0021	II	7	128416520	R
cg22658311	1.27E-04	0.0357	0.0093	II	4	177640279	R
cg27551295	1.31E-04	-0.0099	0.0026	II	17	45894580	F
cg04155280	1.38E-04	-0.0112	0.0029	II	17	26692719	F
cg09055214	1.38E-04	-0.0203	0.0053	II		152915087	R
cg21836827	1.38E-04	0.0153	0.0040	II	6	29720549	F
cg27241576	1.39E-04	-0.0070	0.0018	II	16	81663253	F
cg10171794	1.41E-04	0.0362	0.0094	II	16	10802361	R
cg08170637	1.42E-04	-0.0120	0.0031	II	17	79988088	R
cg14086364	1.42E-04	0.0408	0.0107	II	5	150678334	R
cg19659680	1.45E-04	-0.0189	0.0049	II	11	9385487	F
cg27232569	1.46E-04	-0.0226	0.0059	II	2	232455630	F
cg02328278	1.47E-04	-0.0113	0.0030	II	5	112769927	F
cg03035746	1.47E-04	-0.0944	0.0247	II	2	74727286	R
cg18733798	1.51E-04	0.0289	0.0076	II	14	103469879	F
cg19957032	1.52E-04	0.0573	0.0150	II	14	52455805	R
cg26005361	1.56E-04	-0.0091	0.0024	II	19	42599851	R
cg08079330	1.61E-04	0.0622	0.0164	II	14	76847792	R
cg05236972	1.61E-04	-0.0112	0.0029	I	22	46933233	F
cg12163823	1.64E-04	0.0283	0.0075	I	2	242673721	F
cg06864046	1.65E-04	0.0083	0.0022	II	12	89746505	F
cg05502283	1.66E-04	-0.0105	0.0028	II	19	13203715	R
cg26191661	1.66E-04	-0.0116	0.0031	II	1	16957414	R
cg18530729	1.67E-04	0.0032	0.0008	II	8	30514814	R
cg24830730	1.71E-04	-0.0737	0.0195	I	7	150653195	F
cg09793809	1.73E-04	-0.0096	0.0025	II	1	43352918	R
cg07738268	1.77E-04	-0.0152	0.0040	II	22	46659381	R
cg15734436	1.77E-04	-0.0261	0.0069	II	6	33047185	R
cg13420647	1.80E-04	-0.0640	0.0170	II	1	36883624	F
cg17812026	1.82E-04	-0.0052	0.0014	I	17	1494379	R
cg04462132	1.86E-04	0.1130	0.0301	II	6	161860635	R
cg23474312	1.87E-04	-0.0095	0.0025	II	6	158142707	F
cg18294332	1.87E-04	0.0380	0.0101	II	5	56148274	F
cg25797455	1.90E-04	-0.0113	0.0030	I	7	100064671	R
cg00305285	1.90E-04	-0.0363	0.0097	I	1	1017115	F
cg20248611	1.94E-04	0.0616	0.0164	II	12	34371861	F

cg22495848	1.96E-04	-0.0134	0.0036	I	1	2425173	F
cg24459560	1.97E-04	0.0155	0.0041	II	1	120162965	F
cg06490968	1.98E-04	-0.0442	0.0118	I	16	15974524	R
cg27594095	2.00E-04	-0.0087	0.0023	I	6	31095276	R
cg01930745	2.01E-04	-0.0430	0.0115	II	1	899182	F
cg11068558	2.03E-04	-0.0225	0.0060	I	8	53352237	R
cg15120176	2.04E-04	-0.0092	0.0025	I	18	77142809	F

UCSC_RefGene_Name	UCSC_RefGene_Group	UCSC_CpG_Islands_Name
VAC14	3'UTR	chr16:70719114-707
CSNK1G3;CSNK1G3;CSNK	3'UTR;3'UTR;3	
MYLK2	Body	chr20:30410547-304
PARD6A;ACD;ACD;PARD6	Body;TSS1500;	chr16:67693451-676
SETD1B	Body	chr12:122261329-12
MCF2L;MCF2L	Body;Body	chr13:113701833-11
		chr3:9904331-99048
SAP30BP	Body	
		chr16:2077042-2077
SLC25A10	Body	chr17:79682525-796
TMEM95;TMEM95	5'UTR;1stExon	chr17:7254622-7255
SORCS2	Body	
TCF25	Body	
UCKL1	Body	chr20:62575724-625
MICB	Body	
H2AFY2	Body	
RIC8B	3'UTR	
CD81	TSS1500	chr11:2398223-2399
ANO9	Body	chr11:430814-43177
LSS;LSS;LSS;LSS	3'UTR;3'UTR;3	
IGFBP4	Body	
ZBTB10;ZBTB10	TSS1500;TSS15	chr8:81398429-8139
FAAH	TSS1500	chr1:46859724-4686
KIAA0247	5'UTR	
CPSF1	Body	chr8:145622979-145
		chr14:70700814-707
F12	Body	chr5:176830275-176
BANP;BANP	Body;Body	chr16:88101473-881
FAM125B	Body	
C1orf14;C1orf14	1stExon;5'UTR	chr1:182921819-182
TMEM145	Body	chr19:42817412-428
TMEM175	Body	chr4:940613-941057
INPP5A	Body	chr10:134539817-13
		chr14:106066400-10
WWTR1;WWTR1;WWTR1	Body;Body;Bod	
SLC12A7	Body	chr5:1068746-10690
LOC642006	TSS200	chr7:57247588-5724
DDX20;C1orf183	Body;TSS1500	chr1:112298041-112
THUMPD1	TSS1500	chr16:20752646-207
SLC15A4;MGC16384	Body;TSS200	
SPG7	Body	

FAM116A	1stExon	chr3:57678339-5767
TRAPPC9;TRAPPC9	Body;Body	chr8:140971270-140
		chr1:2435041-24352
CACNA1H;CACNA1H	Body;Body	chr16:1217128-1217
RAI1	5'UTR	chr17:17685016-176
		chr16:88233988-882
TOLLIP	3'UTR	chr11:1298009-1298
RHBDF1;RHBDF1	5'UTR;1stExon	chr16:122017-12287
DFNB31;DFNB31	5'UTR;1stExon	chr9:117266468-117
COASY;COASY;COASY;CO	1stExon;Body;	chr17:40714087-407
MIR665	TSS1500	
OPN1SW	TSS1500	
VEGFC	Body	
OSBPL7	Body	chr17:45890956-458
SEBOX;SEBOX	Body;TSS1500	
DUSP9	Body	chrX:152912491-152
		chr6:29720381-2972
CMIP;CMIP	Body;Body	
RAC3;LRRC45	TSS1500;Body	chr17:79987983-799
SLC36A3;SLC36A3	Body;Body	
		chr11:9385837-9386
TSSK1B;MCC	1stExon;Body	
LBX2	Body	chr2:74725039-7472
CDC42BPB	Body	
C14orf166	TSS1500	chr14:52456128-524
POU2F2	Body	chr19:42599507-426
ESRRB	5'UTR	chr14:76842506-768
CELSR1	TSS200	chr22:46929317-469
D2HGDH	TSS1500	chr2:242673653-242
DUSP6;DUSP6	TSS1500;TSS15	chr12:89745168-897
NFIX	3'UTR	chr19:13207375-132
CROCCL1	TSS200	
GTF2E2	5'UTR	chr8:30515170-3051
KCNH2;KCNH2;KCNH2	Body;Body;TSS	chr7:150655108-150
		chr1:43352785-4335
PKDREJ	TSS200	chr22:46658397-466
HLA-DPB1	Body	chr6:33048416-3304
OSCP1	3'UTR	
SLC43A2	Body	chr17:1492086-1492
PARK2;PARK2;PARK2	Body;Body;Bod	
MAP3K1	Body	
TSC22D4	Body	
		chr1:1016863-10174
		chr12:34371627-343

PLCH2	Body	
ZNF697	3'UTR	chr1:120165302-120
C16orf63	Body	
PSORS1C1	5'UTR	
KLHL17	Body	chr1:894313-902654
		chr18:77138797-771

Relation_to_UCSC_CpG_Island
S_Shore

S_Shelf
S_Shore
N_Shelf

S_Shore
Island

N_Shelf
S_Shore
S_Shelf

Island

N_Shore
Island

N_Shore
N_Shore

S_Shore

S_Shore
Island
N_Shore

Island
Island
S_Shore
N_Shore
Island

S_Shore
Island
Island
S_Shore

Island
Island
S_Shore
N_Shelf
S_Shelf
S_Shore
N_Shore
Island
Island
Island

S_Shelf

S_Shore
Island

Island

N_Shore

S_Shore

N_Shore
Island
S_Shelf
Island
Island
Island
N_Shelf

N_Shore
N_Shore
Island
S_Shore
N_Shore

S_Shelf

Island
Island

N_Shelf

Island

S_Shelf
